# *trans*-Diaqua­(pyridazine-3-carboxyl­ato-κ^2^ *N* ^2^,*O*)lithium

**DOI:** 10.1107/S1600536811000493

**Published:** 2011-01-15

**Authors:** Wojciech Starosta, Janusz Leciejewicz

**Affiliations:** a Institute of Nuclear Chemistry and Technology, ul.Dorodna 16, 03-195 Warszawa, Poland

## Abstract

The structure of the title complex, [Li(C_5_H_3_N_2_O_2_)(H_2_O)_2_], is built of monomeric mol­ecules. In each, an Li^+^ ion is *N*,*O*-chelated by the pyridazine-3-carboxyl­ate ligand and two water O atoms. The coordination geometry of the metal ion is distorted tetra­hedral. The monomers are linked by a system of hydrogen bonds in which water mol­ecules act as donors and carboxyl­ate O atoms act as acceptors. O—H⋯N hydrogen bonding is also present.

## Related literature

For the structures of 3*d* transition metal complexes with the title ligand, see: Ardiwinata *et al.* (1989 ▶); Gryz *et al.* (2003 ▶, 2004 ▶). The structures of complexes with: Mg^2+^ (Gryz *et al.*, 2006 ▶); Ca^2+^ (Starosta & Leciejewicz, 2007 ▶); UO_2_^2+^ (Leciejewicz & Starosta, 2009 ▶) and Pb^2+^ (Starosta & Leciejewicz, 2010 ▶) have been also reported. For the structure of pyridazine-3-carb­oxy­lic acid hydro­chloride, see: Gryz *et al.* (2003 ▶). 
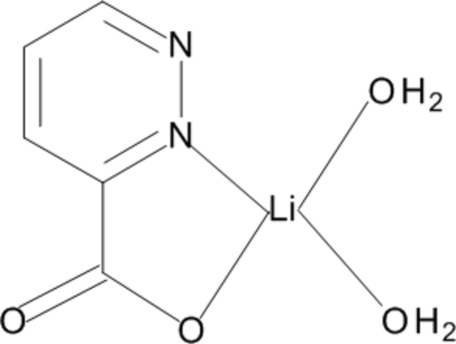


## Experimental

### 

#### Crystal data

[Li(C_5_H_3_N_2_O_2_)(H_2_O)_2_]
*M* *_r_* = 166.07
Monoclinic, 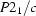

*a* = 7.4620 (15) Å
*b* = 13.738 (3) Å
*c* = 8.0330 (16) Å
β = 112.27 (3)°
*V* = 762.1 (3) Å^3^
*Z* = 4
Mo *K*α radiation
μ = 0.12 mm^−1^
*T* = 293 K
0.41 × 0.13 × 0.11 mm

#### Data collection

Kuma KM-4 four-circle diffractometer
Absorption correction: analytical (*CrysAlis RED*; Oxford Diffraction, 2008) ▶ *T*_min_ = 0.972, *T*_max_ = 0.989
1681 measured reflections
1579 independent reflections
878 reflections with *I* > 2σ(*I*)
*R*_int_ = 0.044
3 standard reflections every 200 reflections  intensity decay: 0.1%

#### Refinement

*R*[*F* ^2^ > 2σ(*F* ^2^)] = 0.047
*wR*(*F* ^2^) = 0.150
*S* = 0.99
1579 reflections
137 parameters
H atoms treated by a mixture of independent and constrained refinement
Δρ_max_ = 0.25 e Å^−3^
Δρ_min_ = −0.30 e Å^−3^

### 

Data collection: *KM-4 Software* (Kuma, 1996 ▶); cell refinement: *KM-4 Software*; data reduction: *DATAPROC* (Kuma, 2001 ▶); program(s) used to solve structure: *SHELXS97* (Sheldrick, 2008 ▶); program(s) used to refine structure: *SHELXL97* (Sheldrick, 2008 ▶); molecular graphics: *SHELXTL* (Sheldrick, 2008 ▶); software used to prepare material for publication: *SHELXTL*.

## Supplementary Material

Crystal structure: contains datablocks I, global. DOI: 10.1107/S1600536811000493/kp2302sup1.cif

Structure factors: contains datablocks I. DOI: 10.1107/S1600536811000493/kp2302Isup2.hkl

Additional supplementary materials:  crystallographic information; 3D view; checkCIF report

## Figures and Tables

**Table 1:** Selected bond lengths (Å)

O1—Li1	1.950 (5)
O3—Li1	1.896 (5)
N2—Li1	2.095 (4)
Li1—O4	1.907 (5)

**Table 2:** Hydrogen-bond geometry (Å, °)

*D*—H⋯*A*	*D*—H	H⋯*A*	*D*⋯*A*	*D*—H⋯*A*
O4—H41⋯O2^i^	0.85 (4)	1.90 (4)	2.720 (3)	164 (3)
O4—H42⋯O2^ii^	0.84 (4)	2.07 (4)	2.823 (3)	150 (4)
O3—H32⋯O1^iii^	0.99 (4)	1.77 (4)	2.741 (3)	167 (3)
O3—H31⋯N1^iv^	0.77 (5)	2.10 (5)	2.840 (3)	160 (5)

Symmetry codes: (i) 
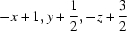
; (ii) 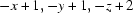; (iii) 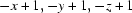; (iv) 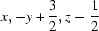.

**Fig. 1.**
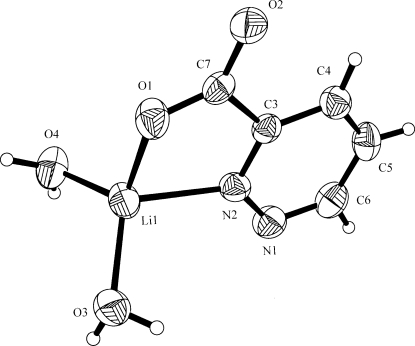
A molecule of the title compound with atom labelling scheme and 50% probability displacement ellipsoids.

**Fig. 2.**
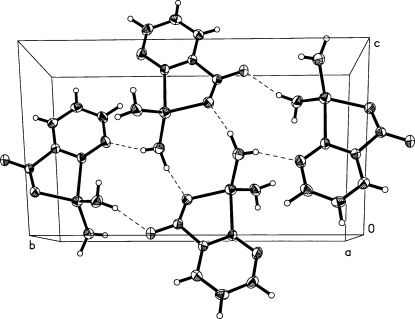
A packing diagram.

## supplementary crystallographic information

### Comment

Metal ion complexes with pyridazine-3-carboxylate ligand show a veriaty of coordination modes and molecular patterns. Monomeric molecules with octahedral coordination geometry have been reported in the structures of a Mn complex (Ardiwinata *et al.* 1989), two Zn complexes (Gryz *et al.*, 2003, 2004) and a Mg complex (Gryz *et al.*, 2006). On the other hand, the structure of a Ca complex is built of binuclear molecules (Starosta & Leciejewicz, 2007). The structure of an uranyl complex is also composed of binuclear molecules but with a different system of internal bridging as compared to that one observed in the crystals of the Ca compound (Leciejewicz & Starosta, 2009). The structure of a Pb complex is catenated polymeric (Starosta & Leciejewicz, 2010). The crystal structure of the title compound contains discrete mononuclear molecules. In each, a Li^1+^ ion is chelated by one pyridazine-3-carboxylate ligand molecule which uses its *N,O*- bonding site and two water O atoms arranged in *trans* mode. The coordination environment of the metal ion is slightly distorted tetrahedral. The relevant Li—O and Li—N bond distances and bond angles are in fair agreement with those reported for Li^1+^ complexes with carboxylate ligands. The pyridazine ring is almost planar [r.m.s. deviation is 0.0099 (1) Å]. The C7/O1/O2 carboxylic group makes with the ring a dihedral angle of 2.0 (1)°. Bond lengths and bond angles within the ligand molecule are close to those reported for the pyridazine-3-carboxylic acid hydrochloride (Gryz *et al.* 2003) and other metal complexes with the title ligand. Coordinated water molecules and carboxylate O atoms participate in a network of hydrogen bonds. An O—H···N of 2.840 (3)%A is also observed. This network is responsible for the stability of the crystal structure.

### Experimental

30 mL of an aqueous solution containing 1 mmol of LiOH (Aldrich) was titrated with 0.1 N HCl until pH of *ca* 6 was reached. Then, a solution of 1 mmol of pyridazine-3-carboxylic acid in 30 mL of hot water was added, the mixture heated at 323 K with stirring for 3 h on a water bath and then left to crystallise at the ambient temperature. After evaporating to dryness well formed single crystals were found on the bottom of the reaction pot. They were washed with ethanol and dried in air.

### Refinement

Water hydrogen atoms were located in a difference map and refined isotropically. H atoms attached to pyridazine-ring C atoms were positioned at calculated positions and treated as riding on the parent atoms, with C—H=0.93 Å and *U*_iso_(H)=1.2*U*_eq_(C).

### Crystal data

**Table:**

[Li(C_5_H_3_N_2_O_2_)(H_2_O)_2_]	*F*(000) = 344
*M**_r_* = 166.07	*D*_x_ = 1.447 Mg m^−^^3^
Monoclinic, *P*2_1_/*c*	Mo *K*α radiation, λ = 0.71073 Å
*a* = 7.4620 (15) Å	Cell parameters from 3 reflections
*b* = 13.738 (3) Å	θ = 6–15°
*c* = 8.0330 (16) Å	µ = 0.12 mm^−^^1^
β = 112.27 (3)°	*T* = 293 K
*V* = 762.1 (3) Å^3^	Blocks, colourless
*Z* = 4	0.41 × 0.13 × 0.11 mm

### Data collection

**Table:**

Kuma KM-4 four-circle diffractometer	878 reflections with *I* > 2σ(*I*)
Radiation source: fine-focus sealed tube	*R*_int_ = 0.044
graphite	θ_max_ = 27.1°, θ_min_ = 3.0°
profile data from ω/2θ scans	*h* = 0→9
Absorption correction: analytical (*CrysAlis RED*; Oxford Diffraction, 2008)	*k* = 0→17
*T*_min_ = 0.972, *T*_max_ = 0.989	*l* = −10→8
1681 measured reflections	3 standard reflections every 200 reflections
1579 independent reflections	intensity decay: 0.1%

### Refinement

**Table:**

Refinement on *F*^2^	Primary atom site location: structure-invariant direct methods
Least-squares matrix: full	Secondary atom site location: difference Fourier map
*R*[*F*^2^ > 2σ(*F*^2^)] = 0.047	Hydrogen site location: inferred from neighbouring sites
*wR*(*F*^2^) = 0.150	H atoms treated by a mixture of independent and constrained refinement
*S* = 0.99	*w* = 1/[σ^2^(*F*_o_^2^) + (0.1002*P*)^2^] where *P* = (*F*_o_^2^ + 2*F*_c_^2^)/3
1579 reflections	(Δ/σ)_max_ < 0.001
137 parameters	Δρ_max_ = 0.25 e Å^−^^3^
0 restraints	Δρ_min_ = −0.30 e Å^−^^3^

### Special details

**Table:**

Geometry. All e.s.d.'s (except the e.s.d. in the dihedral angle between two l.s. planes) are estimated using the full covariance matrix. The cell e.s.d.'s are taken into account individually in the estimation of e.s.d.'s in distances, angles and torsion angles; correlations between e.s.d.'s in cell parameters are only used when they are defined by crystal symmetry. An approximate (isotropic) treatment of cell e.s.d.'s is used for estimating e.s.d.'s involving l.s. planes.
Refinement. Refinement of *F*^2^ against ALL reflections. The weighted *R*-factor *wR* and goodness of fit *S* are based on *F*^2^, conventional *R*-factors *R* are based on *F*, with *F* set to zero for negative *F*^2^. The threshold expression of *F*^2^ > σ(*F*^2^) is used only for calculating *R*-factors(gt) *etc*. and is not relevant to the choice of reflections for refinement. *R*-factors based on *F*^2^ are statistically about twice as large as those based on *F*, and *R*- factors based on ALL data will be even larger.

### Fractional atomic coordinates and isotropic or equivalent isotropic displacement parameters (Å^2^)

**Table:**

	*x*	*y*	*z*	*U*_iso_*/*U*_eq_	
O1	0.5660 (2)	0.46534 (12)	0.7782 (2)	0.0433 (5)	
O2	0.7322 (3)	0.35353 (12)	0.9796 (2)	0.0453 (5)	
O3	0.6476 (3)	0.62905 (16)	0.5350 (3)	0.0504 (5)	
N2	0.7489 (3)	0.60891 (13)	0.9918 (2)	0.0341 (5)	
C3	0.7981 (3)	0.51905 (15)	1.0537 (3)	0.0297 (5)	
N1	0.8297 (3)	0.68561 (14)	1.0952 (3)	0.0436 (5)	
C4	0.9386 (3)	0.5005 (2)	1.2220 (3)	0.0380 (6)	
C6	0.9614 (4)	0.6700 (2)	1.2587 (3)	0.0447 (6)	
C7	0.6887 (3)	0.43841 (16)	0.9250 (3)	0.0331 (5)	
C5	1.0237 (4)	0.5780 (2)	1.3266 (3)	0.0430 (6)	
Li1	0.5577 (6)	0.6038 (3)	0.7221 (5)	0.0411 (9)	
H3	0.974 (4)	0.440 (2)	1.258 (3)	0.043 (7)*	
H6	1.006 (4)	0.728 (2)	1.330 (3)	0.042 (7)*	
H41	0.332 (5)	0.741 (3)	0.668 (4)	0.063 (9)*	
O4	0.3421 (3)	0.68308 (15)	0.7066 (3)	0.0480 (5)	
H42	0.344 (6)	0.691 (3)	0.811 (6)	0.089 (13)*	
H32	0.573 (5)	0.604 (2)	0.413 (5)	0.086 (11)*	
H31	0.678 (6)	0.682 (4)	0.527 (6)	0.108 (17)*	
H5	1.112 (5)	0.571 (2)	1.441 (4)	0.064 (9)*	

### Atomic displacement parameters (Å^2^)

**Table:**

	*U*^11^	*U*^22^	*U*^33^	*U*^12^	*U*^13^	*U*^23^
O1	0.0484 (10)	0.0356 (9)	0.0360 (9)	−0.0082 (8)	0.0048 (8)	−0.0012 (7)
O2	0.0622 (12)	0.0271 (9)	0.0441 (10)	0.0006 (8)	0.0173 (9)	−0.0001 (7)
O3	0.0643 (13)	0.0424 (11)	0.0410 (10)	−0.0133 (10)	0.0161 (10)	−0.0018 (8)
N2	0.0328 (11)	0.0277 (10)	0.0367 (10)	0.0018 (8)	0.0076 (9)	−0.0019 (8)
C3	0.0279 (10)	0.0301 (11)	0.0335 (10)	0.0011 (9)	0.0143 (9)	−0.0013 (9)
N1	0.0438 (12)	0.0338 (11)	0.0462 (12)	−0.0015 (9)	0.0094 (10)	−0.0071 (9)
C4	0.0373 (12)	0.0388 (13)	0.0369 (12)	0.0033 (11)	0.0129 (10)	0.0047 (11)
C6	0.0440 (15)	0.0427 (14)	0.0429 (14)	−0.0128 (12)	0.0114 (12)	−0.0120 (11)
C7	0.0372 (12)	0.0318 (12)	0.0347 (12)	−0.0018 (10)	0.0184 (11)	−0.0014 (9)
C5	0.0333 (13)	0.0566 (15)	0.0328 (13)	−0.0066 (12)	0.0052 (11)	0.0002 (11)
Li1	0.035 (2)	0.043 (2)	0.038 (2)	−0.0004 (18)	0.0061 (18)	0.0022 (17)
O4	0.0532 (12)	0.0418 (11)	0.0478 (12)	0.0100 (9)	0.0177 (9)	0.0099 (9)

### Geometric parameters (Å, °)

**Table:**

O1—C7	1.244 (3)	N1—C6	1.326 (3)
O1—Li1	1.950 (5)	C4—C5	1.357 (4)
O2—C7	1.245 (3)	C4—H3	0.89 (3)
O3—Li1	1.896 (5)	C6—C5	1.386 (4)
O3—H32	0.99 (4)	C6—H6	0.96 (3)
O3—H31	0.77 (5)	C5—H5	0.91 (3)
N2—C3	1.329 (3)	Li1—O4	1.907 (5)
N2—N1	1.336 (3)	Li1—H42	2.31 (4)
N2—Li1	2.095 (4)	O4—H41	0.85 (4)
C3—C4	1.385 (3)	O4—H42	0.84 (4)
C3—C7	1.524 (3)		
			
C7—O1—Li1	117.15 (19)	C4—C5—C6	117.7 (2)
Li1—O3—H32	119 (2)	C4—C5—H5	122 (2)
Li1—O3—H31	117 (3)	C6—C5—H5	120 (2)
H32—O3—H31	109 (4)	O3—Li1—O4	112.8 (2)
C3—N2—N1	120.27 (18)	O3—Li1—O1	111.8 (2)
C3—N2—Li1	109.80 (18)	O4—Li1—O1	121.6 (2)
N1—N2—Li1	129.77 (18)	O3—Li1—N2	120.4 (2)
N2—C3—C4	122.4 (2)	O4—Li1—N2	106.0 (2)
N2—C3—C7	114.86 (19)	O1—Li1—N2	81.06 (16)
C4—C3—C7	122.7 (2)	O3—Li1—C7	117.9 (2)
C6—N1—N2	118.6 (2)	O4—Li1—C7	127.7 (2)
C5—C4—C3	117.6 (2)	O1—Li1—C7	23.74 (9)
C5—C4—H3	121.3 (17)	N2—Li1—C7	57.68 (11)
C3—C4—H3	121.0 (17)	O3—Li1—H42	129.8 (11)
N1—C6—C5	123.3 (2)	O4—Li1—H42	20.1 (10)
N1—C6—H6	114.9 (15)	O1—Li1—H42	113.7 (10)
C5—C6—H6	121.7 (15)	N2—Li1—H42	86.7 (10)
O1—C7—O2	127.8 (2)	C7—Li1—H42	112.3 (11)
O1—C7—C3	116.06 (19)	Li1—O4—H41	121 (2)
O2—C7—C3	116.2 (2)	Li1—O4—H42	108 (3)
O2—C7—Li1	165.72 (18)	H41—O4—H42	102 (3)
C3—C7—Li1	77.30 (15)		

### Hydrogen-bond geometry (Å, °)

**Table:**

*D*—H···*A*	*D*—H	H···*A*	*D*···*A*	*D*—H···*A*
O4—H41···O2^i^	0.85 (4)	1.90 (4)	2.720 (3)	164 (3)
O4—H42···O2^ii^	0.84 (4)	2.07 (4)	2.823 (3)	150 (4)
O3—H32···O1^iii^	0.99 (4)	1.77 (4)	2.741 (3)	167 (3)
O3—H31···N1^iv^	0.77 (5)	2.10 (5)	2.840 (3)	160 (5)

Symmetry codes: (i) −*x*+1, *y*+1/2, −*z*+3/2; (ii) −*x*+1, −*y*+1, −*z*+2; (iii) −*x*+1, −*y*+1, −*z*+1; (iv) *x*, −*y*+3/2, *z*−1/2.
